# Porous Bioactive Prosthesis With Chitosan/Mesoporous Silica Nanoparticles Microspheres Sequentially and Sustainedly Releasing Platelet-Derived Growth Factor-BB and Kartogenin: A New Treatment Strategy for Osteoarticular Lesions

**DOI:** 10.3389/fbioe.2022.839120

**Published:** 2022-02-03

**Authors:** Zhiguo Yuan, Zhuocheng Lyu, Wei Zhang, Jue Zhang, You Wang

**Affiliations:** Department of Bone and Joint Surgery, Department of Orthopaedics, Renji Hospital, School of Medicine, Shanghai Jiaotong University, Shanghai, China

**Keywords:** bioactive scaffold, platelet-derived growth factor-BB, kartogenin, chitosan/mesoporous silica nanoparticles, osteoarticular lesions

## Abstract

Osteochondral lesions represent a major clinical challenge, especially in the elderly. Traditional treatment strategies, such as arthroplasty or tissue engineering, have limitations and drawbacks. In this study, we presented a new treatment concept for the application of an innovative porous bioactive prosthesis with regenerative activity for the treatment of osteoarticular lesions. For regenerative activity, we fabricated chitosan/mesoporous silica nanoparticles (CS/MSNs) composite microspheres *via* the microfluidic method as a dual-factor carrier for the sequential release of platelet-derived growth factor BB (PDGF-BB) and kartogenin (KGN). We then integrated the factor carrier and a nondegradable polyetheretherketone (PEEK) scaffold through a surface modification technique to construct the porous sulfonated PEEK (SPK) @polydopamine (polydopamine)-CS/MSNs scaffold. We systematically evaluated the biocompatibility and biofunctionality of the SPK@PDA-CS/MSNs scaffold and implanted the scaffold in an *in vivo* cartilage defect model in rabbits. These results suggest that the SPK@PDA-CS/MSNs scaffold is biocompatible, promotes cell migration, enhances chondrogenic differentiation of BMSCs *in vitro*, and promotes cartilage regeneration *in vivo*. The porous bioactive prosthesis with regenerative activity presented first in this study may comprise a new therapeutic concept for osteoarticular lesions.

## 1. Introduction

Osteochondral lesions are a common healthcare problem that leads to osteoarticular homeostasis imbalance, joint pain, osteoarthritis, and disability (Bruns et al., 2018; Schreiner et al., 2020). However, as the articular cartilage cannot properly self-repair due to its unique properties of avascularity, no nerves, and low cellularity, osteochondral lesions are a major clinical challenge worldwide (Carballo et al., 2017). In recent years, with advances in surgical and tissue engineering approaches for cartilage/osteochondral lesions and progressive osteoarthritis, different treatment strategies have become available (Bhattacharjee et al., 2015; Kwon et al., 2019). These strategies range from stimulation of biological regeneration to reconstruction techniques through joint surface replacement, depending on disease progression and intervention goal.

Osteoarticular treatment strategies can be divided into two main categories: 1) tissue regeneration stimulation, including microfracture (marrow stimulation) (Frehner and Benthien, 2018), osteochondral autograft/allograft (Chao and Pao, 2017), autologous chondrocyte implantation (ACI) (Krill et al., 2018), matrix-induced autologous chondrocyte implantation (MACI) (Correa Bellido et al., 2019), and autologous matrix-induced chondrogenesis (AMIC) (Tradati et al., 2020); and 2) functional replacement achievement, such as hemiarthroplasty or total joint replacement (Waldorff et al., 2013; Goodman et al., 2020). While these treatment approaches have their own indications, they also have limitations and side effects. For therapeutic strategies aimed at regenerating tissue, the first challenge is the difficulty in achieving complete tissue regeneration, as the regenerated tissue may lack the organization and biomechanics of normal cartilage (Moreira-Teixeira et al., 2011). The second obstacle is the patient’s age, as many regenerative strategies are based on the patients’ own regenerative potential, which is weak in elderly patients. The third challenge is that biological regeneration usually involves complicated procedures (Campos et al., 2019), which also increases the difficulty of clinical application. For functional replacement strategies, the biomechanical and wear characteristics of joint prostheses still have some disadvantages. For example, prosthesis wear problems may result in prosthetic failure due to osteolysis around the implant and fixation loosening (Affatato et al., 2008). Cobalt chromium (CoCr) hemiarthroplasty hip prostheses also fail because of pain and erosion of the acetabular cartilage (Affatato et al., 2008). Consequently, revision surgery is needed, although it is complicated, expensive, and dangerous.

Above all, the goal of complete regeneration for the treatment of osteoarticular lesions is difficult to achieve (Berthiaume et al., 2011), while joint replacement, as a final treatment, is more suitable for end-stage arthritis (Carr et al., 2012). Hence, in the present study, we propose an alternative solution, which is between “regeneration” and “replacement”, for the treatment of cartilage defects in patients who are not good candidates for joint replacement and regenerative procedures.

In our previous unpublished study, we successfully fabricated a porous non-biodegradable polyetheretherketone (PEEK)-based scaffold using 3D printed technology. We observed the physiochemical characteristics and biocompatibility of the scaffold and evaluated its feasibility and safety as a treatment for focal chondral defects in a rabbit model. In that study, we found that the compressive modulus of the 3D printed porous PEEK-based scaffold was close to that of native cartilage, and the porous sulfonated PEEK (SPK) scaffold had excellent cytocompatibility and could be beneficial for cell attachment and proliferation. The *in vivo* study also confirmed that the porous scaffold could promote tissue ingrowth and integration, and achieve cartilage function restoration to a certain extent. Even though the scaffold design was not perfect due to some drawbacks, such as no bioactive stimulator to promote cartilage regeneration, the non-biodegradable porous scaffold still had the potential to be an alternative solution for focal osteochondral lesions, especially functionalizing scaffolds with the capacity for tissue regeneration via surface modification. This can be a new treatment concept for cartilage defects because it neither aims to achieve complete tissue regeneration, nor simple prosthetic replacement, but rather takes an intermediate route between the two. Compared to the traditional treatment strategies of regeneration and replacement, the non-biodegradable porous scaffold with regenerative potential has two main advantages: 1) it can achieve faster recovery of joint function than the regenerative approaches, which require many complicated procedures; and 2) its porous characteristics and regenerative potential may produce a biological articular surface with a low coefficient of friction to avoid or reduce wear problems (Klein, 2009), which are closely associated with prosthetic failure.

**FIGURE1:**
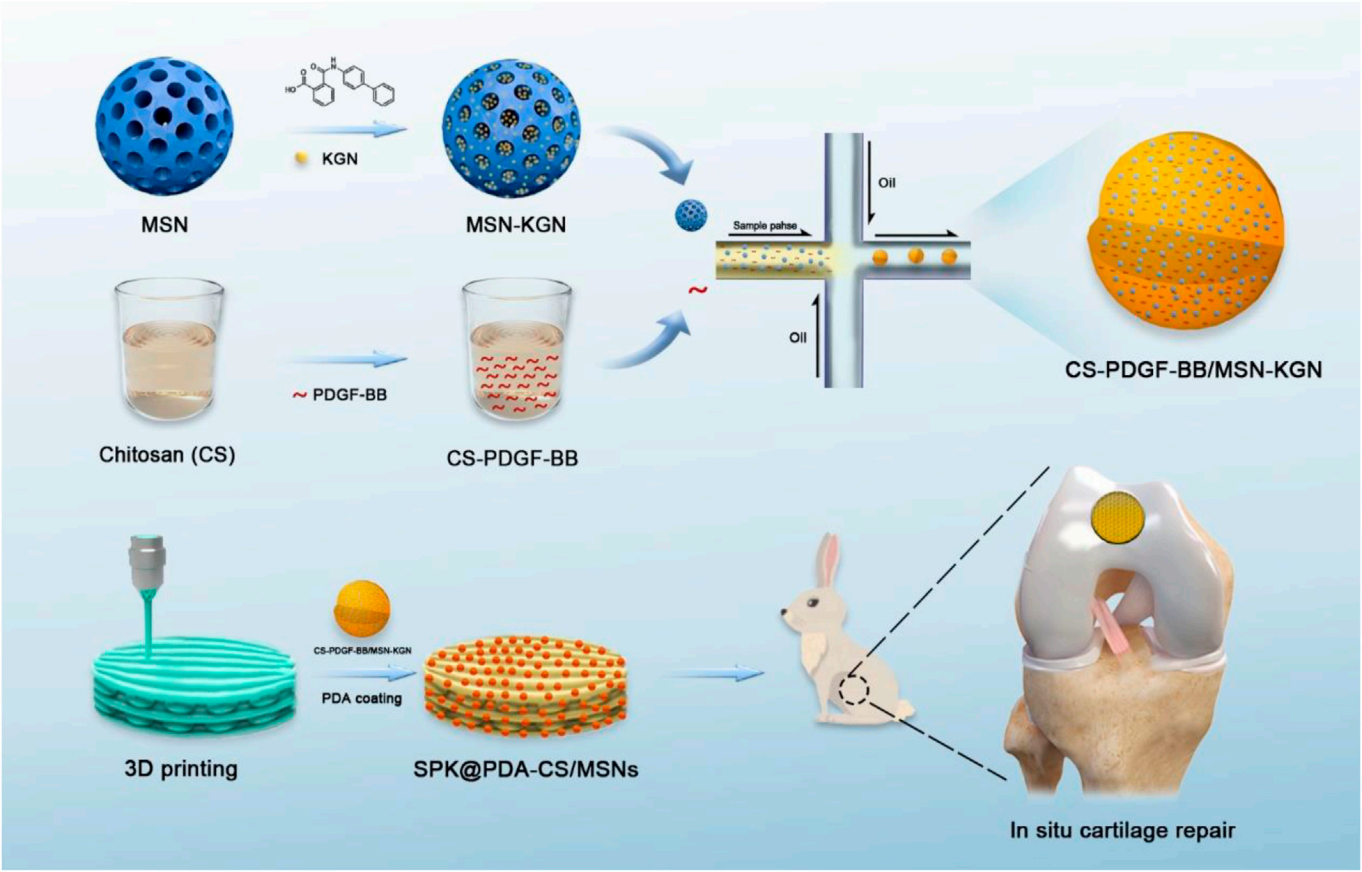
The schematic diagram of this study.

In this study, we fabricated a biocompatible chitosan/mesoporous silica nanoparticles (CS/MSNs) composite microsphere via the microfluidic method as a carrier for dual-factor loading and sequential release of platelet-derived growth factor BB (PDGF-BB) and kartogenin (KGN, a small molecule that promotes chondrogenic differentiation of MSCs). Then, we introduced polydopamine (PDA) into our 3D printed porous PEEK-based scaffold system to immobilize CS/MSNs microspheres onto the pore walls of the porous scaffold with its self-polymerization and adhesion properties. In order to construct a porous bioactive prosthesis, we modified the non-biodegradable porous scaffold with CS/MSNs composite microspheres, which could sequentially release PDGF-BB and KGN, thus stimulating cartilage regeneration. Moreover, we also observed the biocompatibility and biofunctionality of the porous bioactive scaffold and evaluated its feasibility and safety as a treatment approach for focal cartilage defects in a rabbit model (Figure 1).

## 2. Materials and Methods

### 2.1 Preparation and Characterization of Mesoporous Silica Nanoparticles (MSNs)

MSNs were fabricated using the modified Stöber method (Zhao et al., 2015). Briefly, 1.4 g cetyltrimethyl ammonium bromide (CTAB; Macklin, Shanghai, China) and 66 ml deionized water were added to a beaker, which was stirred in an 80°C water bath. After the solution became clear and transparent, 20 ml ethyl acetate (Macklin, Shanghai, China) was added to the beaker, and the reaction was magnetically stirred for 30 min. Then, 14 ml 1 mol/L NH_4_OH was added to the system and the reaction continued under magnetic stirring for 15 min. Next, 7.2 ml tetraethyl orthosilicate (TEOS; Macklin, Shanghai, China) was added and reacted under magnetic stirring for 6 h. Finally, the white porous silica precipitate was collected via centrifugation, washed three times with deionized water and ethanol, dried at 60°C for 12 h, and finally calcined at 700°C for 4 h to obtain MSNs. The morphology and microstructure of MSNs were observed using scanning electron microscopy (SEM; Gemini 300, Zeiss, Germany) and transmission electron microscopy (TEM; Talos F200 X, FEI, Waltham, MA, United States), and the particle size of the MSNs was measured by dynamic light scattering (DLS) using a Zetasizer (Malvern, United Kingdom).

### 2.2 Fabrication of the Microfluidic Device

The microfluidic device is comprised of a microfluidic chip and microfluidic instrumentation. The microfluidic chip (Micronit, Enschede, the Netherlands) was equipped with one inlet and one outlet, main microchannels that were approximately 100 μm in depth and 200 μm in width, and a 50 μm diameter nozzle. Polyethylene tubes with a 0.5 mm inner diameter were inserted as the continuous and sample phase inlets. The microfluidic instrumentation was assembled with a pressure pump (PP), precision pressure controller (PPC), flow sensor (FS), pressure sensor (PS), and liquid storage tank (LST), which were used to pump the fluids into the microfluidic chip (Supplementary Figure S1).

### 2.3 Preparation of CS-MSNs Composite Microspheres

**FIGURE 2:**
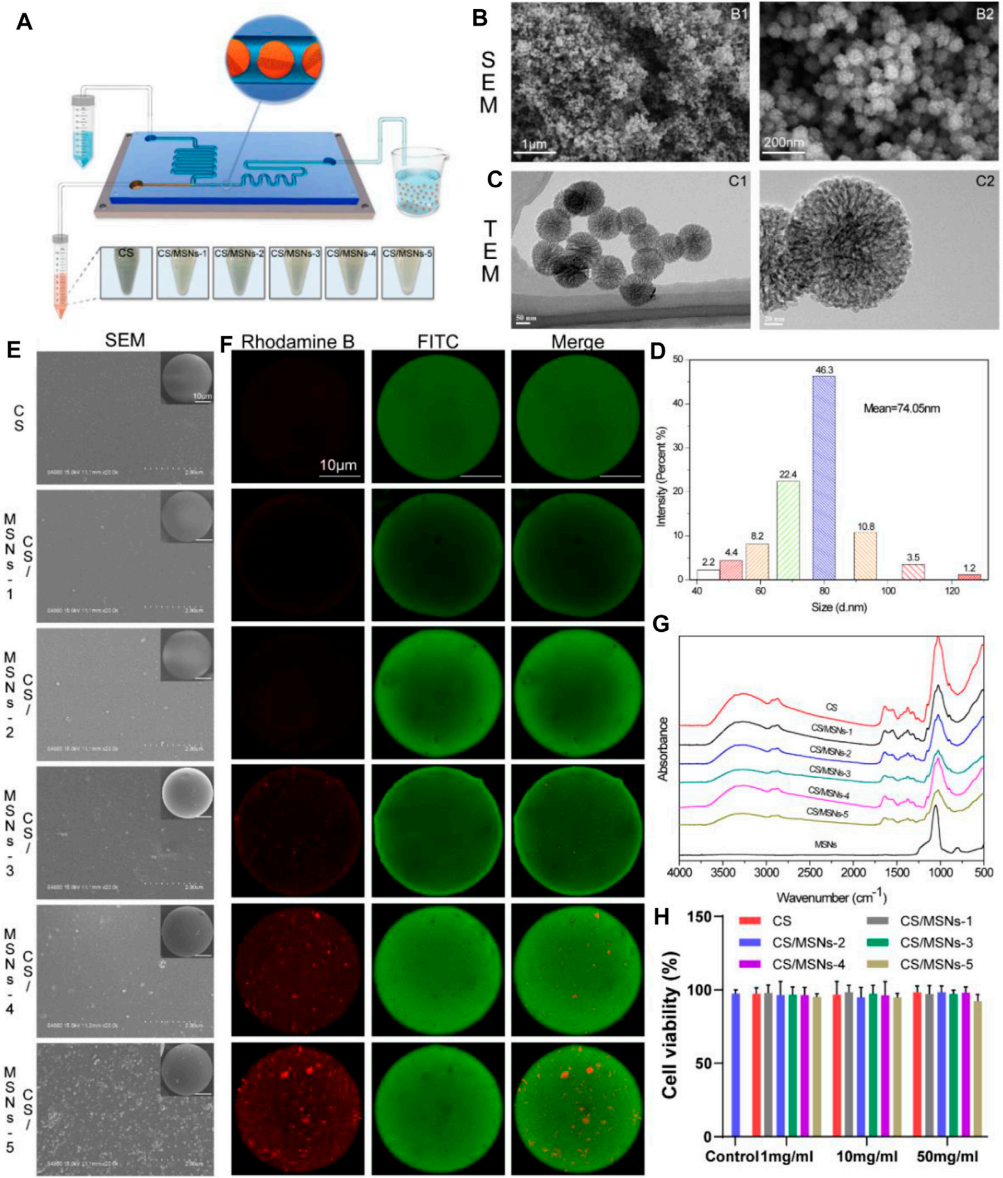
Preparation and characteristics of MSNs and CS/MSNs composite microspheres. **(A)** The schematic illustration of the microfluidic chip for fabrication of CS/MSNs composite microspheres. **(B)** SEM images of MSNs. **(C)** TEM images of MSNs. **(D)** The size distribution of MSNs. **(E)** The SEM images of CS/MSNs composite microspheres. **(F)** The fluorescent images of CS/MSNs composite microspheres. **(G)** FTIR spectra of CS/MSNs composite microspheres. **(H)** Cytotoxicity of different concentrations of CS/MSNs composite microspheres (Data shows mean ± SD, *n* = 5).

An overview of the schedule used to fabricate the CS/MSN-compromised microspheres is shown in Figure 2A. In brief, CS/MSNs microspheres were prepared using a microfluidic approach. First, 200 mg CS (Shanghai Bio Life Science and Technology Co., Ltd, Shanghai, China) was dissolved in 10 ml of a 0.5% acetic acid solution to form a CS solution (2% w/v). Then, 0, 0.5, 1, 5, 10, and 50 mg MSNs were added to a 10 ml chitosan solution to form different CS/MSN mixtures (CS, CS/MSNs-1, CS/MSNs-2, CS/MSNs-3, CS/MSNs-4, CS/MSNs-5), respectively, and the mixtures were ultrasonically oscillated for 10 min and filtered (0.22 μm pore) to obtain a homogeneous solution (Supplementary Figure S2). The CS/MSNs solution, as the sample phase, was set to flow into the microfluidic chip from inlet 2 by the pumps. The oil solution was a 9:1 (v/v) mixture of octane/span 80 and acted as the continuous phase, which was injected into the microchannel from inlet 1 by the pumps, and made the CS aqueous solution form monodispersed droplets under the shear forces. Subsequently, the CS droplets were dripped into cross-linking solutions (2:8 v/v mixture of 2.5% glutaraldehyde/octane) for 30 min to form CS/MSN microspheres. Then, the CS/MSN microspheres were washed twice with deionized water and ethanol. Finally, the CS/MSN microspheres were dried at 37°C under a vacuum.

### 2.4 Characterization of the CS/MSNs Composite Microspheres

The microstructure and surface morphology of the CS/MSN composite microspheres were observed using a scanning electron microscope (SEM, S-4800; Hitachi, Tokyo, Japan). Confocal laser scanning microscopy (CLSM, Leica TCS-SP8; Heidelberg, Germany) was used to evaluate the distribution of MSNs (labeled with rhodamine B) in chitosan (labeled with FITC). The chemical structure of the CS/MSNs composite microspheres was characterized by Fourier transform infrared spectroscopy (FTIR) using a spectrometer (Nicolet Nexus 470; ThermoFisher Scientific, Waltham, MA, United States). Furthermore, X-ray diffraction (XRD) was also used to evaluate the CS/MSNs composite microspheres using an X-ray diffractometer (Ultima IV; Rigaku, Osaka, Japan) in the 2θ range of 5–90°.

### 2.5 *In Vitro* Cytotoxicity Evaluation of the CS-MSNs Composite Microspheres

Rabbit bone marrow mesenchymal stem cells (BMSCs) were isolated, as described in our previous study, and cultured in Dulbecco’s modified Eagle’s medium (DMEM; Gibco, ThermoFisher Scientific, Waltham, MA, United States) with 10% fetal bovine serum (FBS; Gibco, ThermoFisher Scientific, Waltham, MA, United States), penicillin (100 U/mL), and streptomycin (100 μg/ml) at 37°C under a 5% CO_2_ atmosphere.

The *in vitro* cytotoxicity of the CS-MSNs composite microspheres was evaluated on BMSCs using the MTT assay. Briefly, 1 mg/ml, 10 mg/ml, and 50 mg/ml CS and CS/MSNs (CS/MSNs-1, CS/MSNs-2, CS/MSNs-3, CS/MSNs-4, CS/MSNs-5) composite microspheres were incubated in culture medium for 24 h, then the supernatant was filtered using a 0.22 μm membrane (Millex-GP, MilliporeSigma, Burlington, MA, United States). BMSCs with a density of 5 × 10^3^ cells per well were seeded in 96-well plates and incubated for 24 h. Then the cells were treated with the extracts of different concentrations of CS and CS/MSNs composite microspheres for another 24 h. Subsequently, the cells were stained using the MTT assay, and the OD values were measured at 570 nm using a microplate reader (Bio-Rad Model 550; Hercules, CA, United States). Cell viability (%) was calculated as [OD]_sample_/[OD]_control_ × 100.

### 2.6 PDGF-BB and KGN Loading and *In Vitro* Release Study

To prepare the CS microspheres loaded with PDGF-BB, a 10 ml CS aqueous solution with 200 mg CS (2% wt) and 1 mg PDGF-BB (100 ng/ml) was used, and the fabrication procedures were performed as described above. In the case of MSNs loaded with KGN, 10 mg MSNs were added to 1 ml of 1 μM KGN PBS solution, ultrasonicated for 5 min, and then stirred for 24 h to enable the KGN to fully impregnate the MSNs. Subsequently, MSNs-KGN was obtained by centrifugation at 15,000 rpm for 10 min. For the preparation of CS-PDGF-BB/MSNs-KGN composite microspheres, 10 mg MSNs-KGN was added to a 10 ml CS aqueous solution with 200 mg CS (2 wt%) and 1 mg PDGF-BB (100 ng/ml), and subjected to ultrasonic oscillation for 10 min to obtain a homogeneous CS/MSNs solution. The fabrication procedures of the CS-PDGF-BB/MSNs-KGN composite microspheres were performed as described above.

The *in vitro* release profiles of PDGF-BB and KGN from the CS-PDGF-BB/MSNs-KGN microspheres were compared with those of the CS-PDGF-BB microspheres and MSNs-KGN in PBS at 37°C. CS-PDGF-BB/MSNs-KGN microspheres (5 mg), CS-PDGF-BB microspheres (5 mg), and MSNs-KGN (5 mg) were added to 50 ml PBS, and the mixture was shaken at 100 rpm. At the set time intervals, 100 μl supernatant was extracted from each sample and then added to 100 μl of fresh PBS. The PDGF-BB concentration in the supernatant was quantified using a PDGF-BB ELISA Kit (Neobioscience, Shenzhen, China), and the KGN concentration was quantified by HPLC.

### 2.7 Preparation of the 3D Printed Porous PEEK Scaffold and SPK@PDA-CS/MSNs Scaffold

The PEEK scaffolds were fabricated using a fused filament fabrication (FFF) 3D printer, according to our previous study. The printing parameters of the scaffold, such as strut size (nozzle size), pore size, layer height, and dimensions, were set to the following: 250 μm strut size, 400 μm pore size, 1 mm layer height, and Ø4 mm x 1 mm dimensions.

The sulfonated PEEK scaffold (SPK) was fabricated as follows. The PEEK scaffolds were treated with concentrated sulfuric acid (98 wt%), subjected to ultrasonic oscillation for 30 s, and then washed with deionized water for 6 h.

CS/MSNs composite microspheres (containing PDGF-BB and KGN) were immobilized in the SPK scaffold using the PDA coating method. The SPK scaffolds were immersed in Tris-HCl buffered solution (pH 8.5) with 2 mg/ml dopamine and 20 mg/ml CS/MSNs microspheres (containing PDGF-BB and KGN) under magnetic stirring for 4 h (termed SPK@PDA-CS/MSNs), and the samples without CS/MSNs microspheres were denoted as SPK@PDA.

### 2.8 Characterization of SPK@PDA-CS/MSNs Scaffold

#### 2.8.1 Scanning Electron Microscopy (SEM)

The surface morphologies of PEEK, SPK, SPK@PDA, and SPK@PDA-CS/MSNs scaffolds were observed using an emission scanning electron microscope (SEM, Hitachi S-4800; Hitachi, Tokyo, Japan). The prepared specimens were dried and coated with gold prior to the SEM observation.

#### 2.8.2 Hydrophilic Property

We used the water contact angle (WCA) to evaluate the surface hydrophilicity of the scaffolds via a contact angle goniometer (JY-82B; Kruss DSA, Hamburg, Germany) at room temperature. Deionized water (2 μl) was dropped on the surface of the samples, left for 3 s, and observed using a video contact angle system. Five repeated measurements were performed on each specimen to provide an average.

### 2.9 Biocompatibility of the SPK@PDA-CS/MSNs Scaffold

#### 2.9.1 Cell Adhesion

Rabbit BMSCs were obtained and cultured as described previously. Each of these PEEK, SPK, SPK@PDA, and SPK@PDA-CS/MSNs scaffolds were seeded with 5×10^4^ cells. After culturing for 6, 12, and 24 h, the cell-scaffold complexes were collected. The cells in the scaffold were measured using a Cell Counting Kit-8 (CCK-8, Beyotime, Shanghai, China) assay, and the OD values of these specimens were measured at 450 nm using a microplate reader (Synergy HT; Bio-Tek Co., Winooski, VT, United States).

#### 2.9.2 Live/Dead Staining

The cytocompatibility of the PEEK, SPK@PDA, and SPK@PDA-CS/MSNs scaffolds was assessed using a live/dead assay kit (Beyotime, Shanghai, China) according to the manufacturer’s instructions. After 3 and 7 days of culture, the cell/scaffold complex was collected and washed with phosphate buffered saline (PBS) twice, and then treated with calcein AM and propidium iodide (PI) (Beyotime) for 0.5 h. Subsequently, the specimens were washed with PBS twice and then observed using a fluorescence confocal microscope (Leica SP8, Weztlar, Germany). Furthermore, all procedures were performed in a dark room.

#### 2.9.3 Cytoskeletal Observations

The morphology of cells on the PEEK, SPK@PDA, and SPK@PDA-CS/MSNs scaffolds was evaluated by cytoskeletal observations using F-actin staining. After 3 days of culture, cells on different samples were fixed with paraformaldehyde (4% v/v) for 30 min, washed twice with PBS, and stained with FITC-phalloidin (Solarbio, Beijing, China) for 40 min. After washing with PBS twice, the cells were treated with 4’,6-diamidino-2-phenylindole (DAPI; Beyotime, Shanghai, China) for 10 min, and then observed using a Leica SP8 fluorescence confocal microscope.

### 2.10 I*n Vitro* Cell Migration Evaluation of the SPK@PDA-CS/MSNs Scaffold

We used the transwell chemotactic migration system (Corning, Corning, NY, United States) to evaluate the recruitment ability of PDGF-BB released from the SPK@PDA-CS/MSNs scaffold. In brief, 5 × 10^4^ BMSCs were seeded in the upper chamber of the transwell plate, and the SPK@PDA and SPK@PDA-CS/MSNs scaffolds were placed in the lower chamber. After 24 h of culture, the transwell membrane was scraped on the upper surface to remove the cells, and the migrated cells on the lower side were fixed in 4% paraformaldehyde, stained with crystal violet, imaged using an optical microscope (Olympus Corporation, Japan), and quantitatively evaluated using the Imaris software.

### 2.11 Chondrogenic Differentiation Evaluation of the SPK@PDA-CS/MSNs Scaffold

#### 2.11.1 Chondrogenic Differentiation in BMSCs Pellets

We evaluated the chondrogenic differentiation capacity of KGN released from the SPK@PDA-CS/MSNs scaffold using a transwell system (Corning, NY, United States). First, 5×10^5^ BMSCs were placed into a 15 ml centrifuge tube, centrifuged at 250 g for 5 min, and then cultured for 2 days to form BMSC pellets. The same transwell plate used in the cell migration experiment was also used in the chondrogenic differentiation experiment. The pellets were transferred to the fresh upper well of the transwell plate, and the SPK@PDA-CS/MSNs scaffold was positioned in the lower well. The chondrogenic culture medium (Cyagen, Santa Clara, CA, United States) in the transwell plate was exchanged every 3 days. After 21 days of culture, the pellets were photographed and analyzed.

#### 2.11.2 Histological and Immunohistochemical Examination

After culturing for 21 days, the pellets were harvested, fixed in 4% paraformaldehyde for 2 days, dehydrated, embedded, and sectioned into 7 μm slices. These sections were stained with hematoxylin-eosin (HE), Alcian blue (AB), and Safranin O/Fast Green (SO/FG). The sections were then subjected to immunohistochemical (IHC) analysis for collagen II (primary antibody Col II 1:100; Abcam, Boston, MA, United States). Immunohistochemical staining was performed as described in our previous study (Yuan et al., 2021).

#### 2.11.3 GAG/DNA

After 21 days of culture, the pellets were harvested and assayed for DNA and GAG content. The DNA of the pellets was extracted using a genomic DNA kit (TIANamp, Beijing, China), and then quantified using a PicoGreen DNA assay kit (Invitrogen, Carlsbad, CA, United States). The GAG content of the pellets was measured using a tissue GAG total content 1,9-dimethylmethylene blue (DMMB) colorimetric kit (Genmed Scientific Inc, Shanghai, China). The GAG secreted by the cells was standardized using GAG/DNA.

#### 2.11.4 RT-PCR

We evaluated the expression of cartilage-related genes SRY-Box Transcription Factor 9 (SOX9), aggrecan, collagen I, and collagen II in the pellets of the SPK@PDA-CS/MSNs and SPK@PDA groups using real-time polymerase chain reaction (RT-PCR) experiments. In brief, RNA was extracted using TRIzol (Sigma-Aldrich, St. Louis, MO, United States) and transcribed into complementary DNA (cDNA) using a ReverTra Ace kit (Toyobo, Osaka, Japan). Gene expression was quantified by quantitative reverse transcription polymerase chain reaction (qRT-PCR) using a LightCycler 480 system (Roche Applied Science, Indianapolis, IN, United States). The primers used in this study are shown in Supplementary Table S1, and glyceraldehyde-3-phosphate dehydrogenase (GAPDH) was used as a reference gene.

### 2.12 *In Vivo* Animal Studies

#### 2.12.1 Surgical Procedure

Animal studies were approved by the Institutional Animal Care and Use Committee of Renji Hospital, which was affiliated with Shanghai Jiao Tong University Medical College. Twenty-four adult New Zealand white rabbits were used in this study and were randomly allocated into three groups: SPK@PDA-CS/MSNs, SPK@PDA, and the control group. A Ø4 mm × 1 mm cylinder defect was fashioned using a punch in the femoral trochlear to create a cartilage defect model of the rabbits. In the SPK@PDA-CS/MSNs group, the defect was treated with the SPK@PDA-CS/MSNs scaffold (containing PDGF-BB and KGN), whereas in the SPK@PDA group, the defect was treated with the SPK@PDA scaffold. The defect was left untreated in the control group. Postoperatively, the rabbits were administered antibiotics to prevent infection and were left free to move in the cages. The rabbits were euthanized at 1 and 3 months after surgery and were prepared for analysis.

#### 2.12.2 Macroscopic Observations

The femoral condyles were harvested and photographed at 1 and 3 months after surgery. The macroscopic observations in the different groups were semi-quantitatively analyzed via the macroscopic scoring system from Goebel et al. (Goebel et al., 2012; Madry et al., 2020), which was performed by two blinded experienced investigators. The scoring details were presented in Supplementary Table S2.

### 2.12.3 Histological Examination

The femoral condyles were harvested at 1 and 3 months postoperatively, fixed in 4% paraformaldehyde for 2 days, decalcified with ethylenediaminetetraacetic acid (EDTA, Servicebio, Wuhan, Hubei, China), dehydrated, embedded, and sectioned into 7 μm slices. The sections were stained with HE and toluidine blue (TB). The sections were also subjected to IHC staining for collagen II as described above.

#### 2.12.4 Biochemical Assays for GAG and Collagen

We evaluated the GAG content of the repaired tissue using a tissue GAG total content DMMB colorimetric kit (GenMed, Shanghai, China). The total collagen content of the repaired tissue was measured using a hydroxyproline assay kit (Nanjing Jiancheng Bioengineering Institute, Nanjing, China) according to the manufacturer’s instructions.

#### 2.12.5 RT-PCR

To further evaluate cartilage regeneration, we used RT-PCR to evaluate the expression of the cartilage-related genes SOX9, aggrecan, collagen I, and collagen II. RNA was extracted, reverse-transcribed, and quantified, as described above. The primer sequences are listed in Supplementary Table S1, and GAPDH was used as an internal control.

### 2.13 Statistical Analysis

Statistical analysis was performed with Tukey’s multiple comparison tests, as well as one-way analysis of variance (ANOVA), using GraphPad Prism 9 (GraphPad Software, Inc, La Jolla, CA, United States). All data were expressed as the mean ± standard deviation (SD), and the significance of the difference was set at *p* < .05.

## 3. Results

### 3.1 Characterization of MSNs and CS/MSNs Composite Microspheres

As shown in Figure 2B, the morphology of the MSNs was observed using SEM. It could be seen that the MSNs were nanoscale particles. The internal pore structure of the MSNs was observed using TEM. We confirmed that the MSNs were monodisperse and possessed a highly porous structure and sufficient space for the adsorption of biomolecules, such as KGN (Figure 2C). The particle size distribution of the MSNs was measured using dynamic light scattering (DLS), as shown in Figure 2D. The particle size was approximately 74 nm, and it was mainly distributed between 68 and 92 nm.

Moreover, different amounts of MSNs (0.5, 1, 5, 10, and 50 mg) were added to 10 ml chitosan solution, and then ultrasonically oscillated for 10 min to form a homogeneous CS/MSNs (CS/MSNs-1, CS/MSNs-2, CS/MSNs-3, CS/MSNs-4, and CS/MSNs-5) mixture solution. As shown in Supplementary Figure S2, CS/MSNs-1, CS/MSNs-2, CS/MSNs-3, and CS/MSNs-4 mixture solutions were homogeneous, while there were some sediments in the CS/MSNs-5 mixture solution, indicating that 50 mg MSNs cannot disperse completely in a 10 ml chitosan solution. Next, different CS/MSN composite microspheres were prepared from a chitosan mixed solution by the microfluidic method.

Subsequently, the CS/MSN composite microspheres were characterized by SEM, fluorescence labeling, FTIR, and XRD. As shown in Figure 2E, SEM was used to observe the surface morphology of the CS/MSN composite microspheres. It can be seen that the structures of the CS/MSNs composite microspheres were significantly different, and the distribution of the MSNs in the CS/MSNs composite microspheres increased as the MSNs concentration increased. Furthermore, we utilized fluorescence labeling (MSNs labeled with rhodamine B, and CS marked with FITC) to observe the internal structure of CS/MSNs composite microspheres, as shown in Figure 2F. The CLSM results showed that the MSN content in the CS/MSNs composite microspheres increased with the addition of MSNs, and MSNs aggregation was evident in the CS/MSNs-5 group, which was consistent with the results of SEM.

The main chemical groups of CS microspheres, CS/MSNs composite microspheres, and MSNs could be evaluated from the FTIR spectra, as shown in Figure 2G. For the CS microspheres, the characteristic peaks were at 2,872 cm^−1^ (C-H stretching vibration), 1,634 cm^−1^ (-C=O stretching vibration), 1,550 cm^−1^ (N-H stretching vibration), and 1,024 cm^−1^ (C-O stretching vibration). The characteristic absorption peak of MSNs at 806 cm^−1^ (Si-O-Si stretching vibration) was weakened in the spectra of the CS/MSNs composite microspheres, which may mean that the MSNs were dispersed into the CS matrix. For the CS/MSNs composite microspheres, the main characteristic peaks were also observed at 2,872 cm^−1^, 1,634 cm^−1^, 1,550 cm^−1^, and 1,024 cm^−1^, which were similar to those of the CS microspheres. Furthermore, the CS microspheres, CS/MSNs composite microspheres, and MSNs were characterized by XRD, as shown in Supplementary Figure S3. The characteristic diffraction peak of the CS microspheres was at 2θ = 19.94° (hydrated crystalline). MSNs appeared as an intense reflection peak at 2θ = 21.6°. With the addition of the MSNs, the characteristic reflection peak of the CS/MSNs composite microspheres was similar to that of the CS microspheres, indicating that the MSNs were dispersed in the CS matrix.

Next, we assessed the cytotoxicity of the CS microspheres and CS/MSNs composite microspheres at 1 mg/ml, 10 mg/ml, and 50 mg/ml using BMSCs by MTT assay. Figure 2H shows the cell viability of BMSCs treated with the extracts of CS microspheres and CS/MSNs composite microspheres at 1 mg/ml, 10 mg/ml, and 50 mg/ml, respectively. The cell viability of CS/MSNs composite microspheres was approximately the same as that of the CS microspheres and the control group; the cell viability of the different samples was above 90%, indicating that CS and CS/MSNs would be safe as drug delivery carriers. As for the five different CS/MSNs composite microspheres (CS/MSNs-1, CS/MSNs-2, CS/MSNs-3, CS/MSNs-4, and CS/MSNs-5), the SEM and CLSM results also confirmed that, with the addition of the MSNs, the distribution of the MSNs in the CS/MSNs composite microspheres increased. In contrast, in CS/MSNs-5, the MSNs aggregation could be observed. Similarly, the CS/MSNs-5 mixture solution contained some sediments, indicating that 50 mg MSNs could not disperse completely in a 10 ml chitosan solution. Therefore, we chose CS/MSNS-4 as a drug delivery carrier.

### 3.2 *In Vitro* Release of PDGF-BB and KGN

**FIGURE 3:**
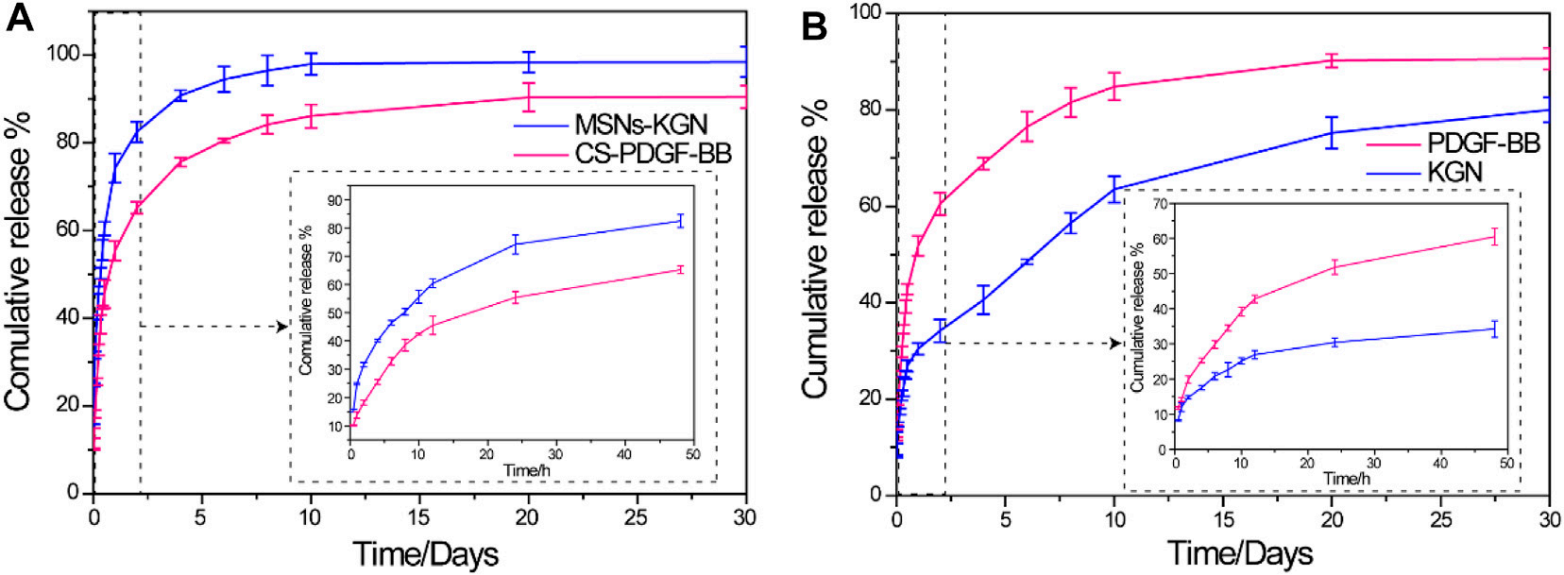
**(A)** *In vitro* release profiles of KGN from MSNs and PDGF-BB from CS in PBS solution. **(B)** *In vitro* release profiles of PDGF-BB and KGN from CS/MSNs composite microspheres. Date was shown as mean ± SD, *n* = 3.

The PDGF-BB and KGN release profiles of the MSNs loaded with KGN, CS microspheres loaded with PDGF-BB, and CS/MSNs composite microspheres loaded with PDGF-BB and KGN *in vitro* were investigated using ELISA kits and HPLC (Figure 3). For the KGN release from the MSNs, which was showed in Figure 3A, there was a rapid release of 74% of the total KGN released from the MSNs on the first day, after which the release rate slowed and reached approximately 98% of the total KGN released after 10 days. For the PDGF-BB release from the CS microspheres loaded with PDGF-BB, there was still an initial burst release, with nearly 55% of the total PDGF-BB released on the first day; subsequently, the release slowed and approximately 86% was released after 10 days. In contrast, the drug release behavior of the composite microspheres was completely different from that of the pure MSNs and CS microspheres, as shown in Figure 3B. The release of KGN from CS/MSNs composite microspheres (loaded with PDGF-BB and KGN) was sustained and controlled, with a comparatively slow release of 30% on the first day, and then sustainably released for as long as 3 weeks. The PDGF-BB release from CS/MSNs composite microspheres was sharp compared with KGN, with 51% released on the first day, and nearly 84% released at 10 days, whereas approximately 40% of the total KGN was retained after 10 days. The results indicated that PDGF-BB and KGN were sequentially released from the CS/MSNs composite microspheres. According to the literatures (Charnay et al., 2004; Zhao et al., 2015), drugs (KGN in this study) were loaded in the pore walls of the MSNs and were usually located in the center of the MSNs. As for the CS/MSNs composite microspheres, the KGN first traveled to the surface of the MSNs and entered the CS matrix, then passed through the CS matrix, lengthening the diffusion route, whereas the PDGF-BB diffused directly out of the CS matrix. Therefore, the sequential release behavior of PDGF-BB and KGN from the CS/MSNs composite microspheres could be attributed to the characteristic composite structure of the CS/MSNs.

### 3.3 Characterization of the SPK@PDA-CS/MSNs Scaffold

**FIGURE 4:**
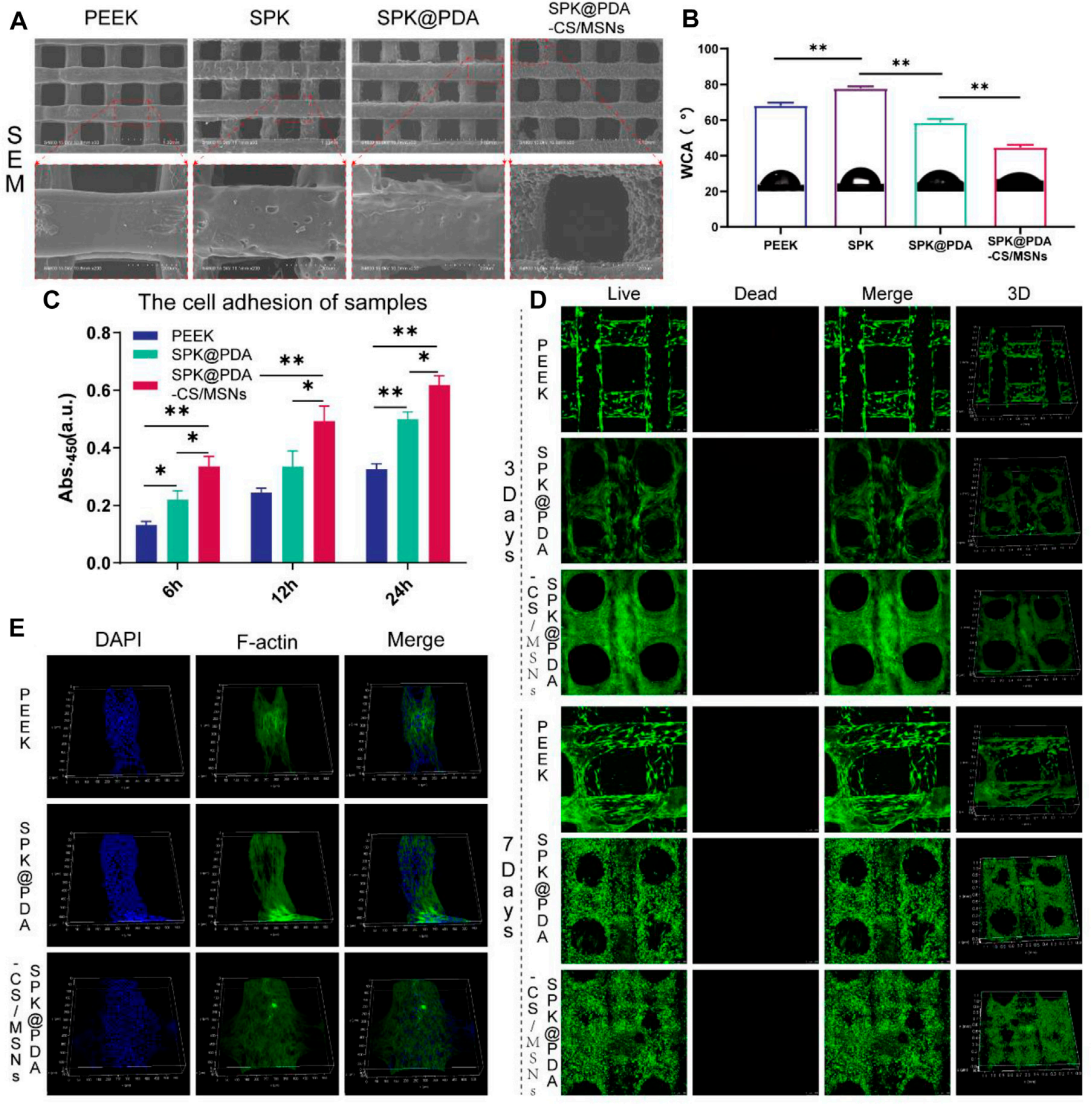
The properties and cytocompatibility of the prepared scaffolds. **(A)** The SEM images of the PEEK, SPK, SPK@PDA, and SPK@PDA-CS/MSNs scaffolds. **(B)** The water contact angle (WCA) of the prepared specimens. **(C)** The cell adhesion of the PEEK, SPK@PDA, and SPK@PDA-CS/MSNs scaffolds. Data are presented as mean ± SD, *n* = 5, *p < .05, **p < .01. **(D)** Live/Dead staining. **(E)** Three-dimensional fluorescence images of BMSCs on the scaffolds for 3 days of culture.

We evaluated the microstructure and hydrophilicity of the PEEK, SPK, SPK@PDA, and SPK@PDA-CS/MSNs scaffolds using SEM and the water contact angle (WCA). As shown in Figure 4A, Supplementary Figure S4, the morphology observation of the different scaffolds was conducted using general observation and SEM. The PEEK scaffold had a smooth surface, while the SPK and SPK@PDA scaffolds were rougher, which was mainly attributed to the sulfonation treatment and PDA coating. The SEM results also confirmed that the CS/MSNs composite microspheres could be immobilized in the SPK@PDA-CS/MSNs scaffold by the PDA coating method. Figure 4B shows the water angles of the prepared scaffolds and confirmed that these surface modification methods altered the surface wettability of the scaffolds. The sulfonation treatment changed the hydrophilia of the PEEK scaffold from 68.2 ± 1.5°(PEEK) to 77.9 ± 1.1°(SPK), whereas the PDA coating enhanced the hydrophilicity of SPK from 77.9 ± 1.1° to 58.6 ± 1.9°. After the deposition of CS/MSNs composite microspheres in the scaffold, the contact water angles further decreased to 44.7 ± 1.4°.

### 3.4 Cytocompatibility of the SPK@PDA-CS/MSNs Scaffold

The cytocompatibility of the prepared specimens, including the PEEK, SPK@PDA, and SPK@PDA-CS/MSNs scaffolds, was evaluated via a cell adhesion assay, live/dead staining, and cytoskeletal staining. The cell adhesion of the scaffolds was evaluated using the CCK-8 kit, and the results in Figure 4C demonstrate that the cell attachment capacity of the SPK@PDA-CS/MSNs scaffold was superior to that of the PEEK and SPK@PDA scaffolds. We then assessed the cell growth of the BMSCs on the PEEK, SPK@PDA, and SPK@PDA-CS/MSNs scaffolds through live/dead staining after culturing them for 3 and 7 days. As shown in Figure 4D, most of the BMSCs on the three different scaffolds were alive after 3 and 7-days culture, and there was an increase in cells on the scaffolds as the culture time increased. In addition, there were more cells on the SPK@PDA-CS/MSNs scaffolds than that on the PEEK and SPK@PDA scaffolds. Furthermore, we observed the morphology of the cells on the three scaffolds after 3-days culture through cytoskeletal staining, which is shown in Figure 4E, Supplementary Figure S5. The BMSCs were spread well on all three scaffolds, while there were more well-spread cells on the SPK@PDA-CS/MSNs scaffold compared to the PEEK and SPK@PDA scaffolds. Therefore, all the experiments demonstrated that the SPK@PDA-CS/MSNs scaffold had superior cytocompatibility and biocompatibility.

### 3.5 *In Vitro* Cell Migration

**FIGURE 5:**
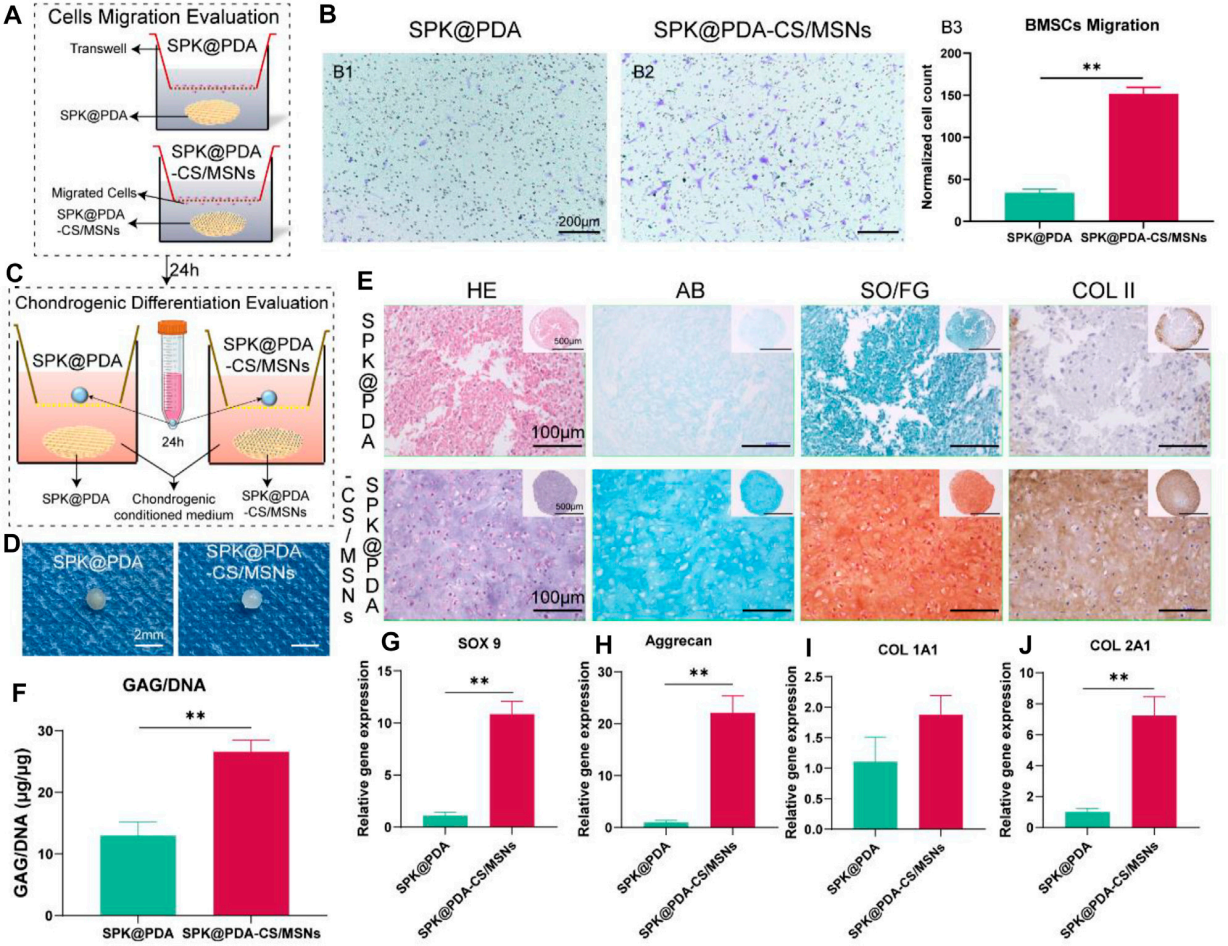
*In vitro* cell migration and chondrogenic differentiation evaluation of the SPK@PDA and SPK@PDA-CS/MSNs scaffolds. **(A)** Schematic illustration of the transwell cell migration model. **(B)** Cell migration assay of the scaffolds showed the representative images of migrated cells in the SPK@PDA (B1) and SPK@PDA-CS/MSNs (B2) groups, and the quantitative evaluation of migrated cells in different groups (B3). Data are shown as mean ± SD, *n* = 4. **p < .01. **(C)** The transwell system was used to evaluate the chondrogenic differentiation effect of the SPK@PDA and SPK@PDA-CS/MSNs scaffolds. **(D)** Gross observation of the pellets treated with SPK@PDA and SPK@PDA-CS/MSNs scaffolds. **(E)** Histologic analysis of the pellets. **(F)** GAG normalized by DNA of the pellets. Data are expressed as mean ± SD (*n* = 3; **p < .01). G-J. Expression of cartilage-related genes SOX9 **(G)**, aggrecan **(H)**, collagen I **(I)**, and collagen II **(J)** of the pellets through RT-PCR. Data are shown as mean ± SD, *n* = 3. **p < .01.

The chemotactic capacity of the SPK@PDA-CS/MSNs scaffold (containing PDGF-BB and KGN) was evaluated *via* a transwell system, as shown in Figure 5A. The BMSCs traversing the transwell membrane were identified and quantified by crystal violet staining, and the results are shown in Figure 5B. The number of migrating cells in the SPK@PDA-CS/MSNs group was significantly higher than that in the SPK@PDA group, confirming the recruitment capacity of PDGF-BB released from the SPK@PDA-CS/MSNs scaffold.

### 3.6 *In Vitro* Chondrogenic Differentiation Evaluation

We evaluated the chondrogenic differentiation capacity of the SPK@PDA and SPK@PDA-CS/MSNs scaffolds in the BMSC pellet culture using the transwell system (Figure 5C). As shown in Figures 5D,E, the pellet treated with SPK@PDA-CS/MSNs after a 21-days culture was more transparent than that in the SPK@PDA group. The HE results showed that there were plenty of chondrocyte-like cells and cartilage lacuna in the pellet treated with SPK@PDA-CS/MSNs, whereas the SPK@PDA-treated pellet showed abundant spindle-shaped cells and a loose structure. The AB and SO/FG staining showed greater intensity in the pellets treated with SPK@PDA-CS/MSNs than in those treated with SPK@PDA. Moreover, the GAG per DNA content of the pellet treated with SPK@PDA-CS/MSNs increased significantly compared with that of the SPK@PDA-treated pellet, which was consistent with the histological results (Figure 5F). We then evaluated the expression of chondrogenic differentiation-related genes, including SOX 9 (Figure 5G), aggrecan (Figure 5H), collagen I (Figure 5I), and collagen II (Figure 5J), in the pellets treated with SPK@PDA and SPK@PDA-CS/MSNs by RT-PCR. The expression of SOX 9, aggrecan, and collagen II increased significantly in the pellet treated with SPK@PDA-CS/MSNs compared to those treated with SPK@PDA, while only slight changes were observed in the collagen I expression of the pellets treated with SPK@PDA and SPK@PDA-CS/MSNs, with no statistical significance. These results demonstrated that the chondrogenic differentiation capacity of the BMSC pellets was enhanced by treatment with SPK@PDA-CS/MSNs.

### 3.7 *In Vivo* Cartilage Repair Studies

**FIGURE 6:**
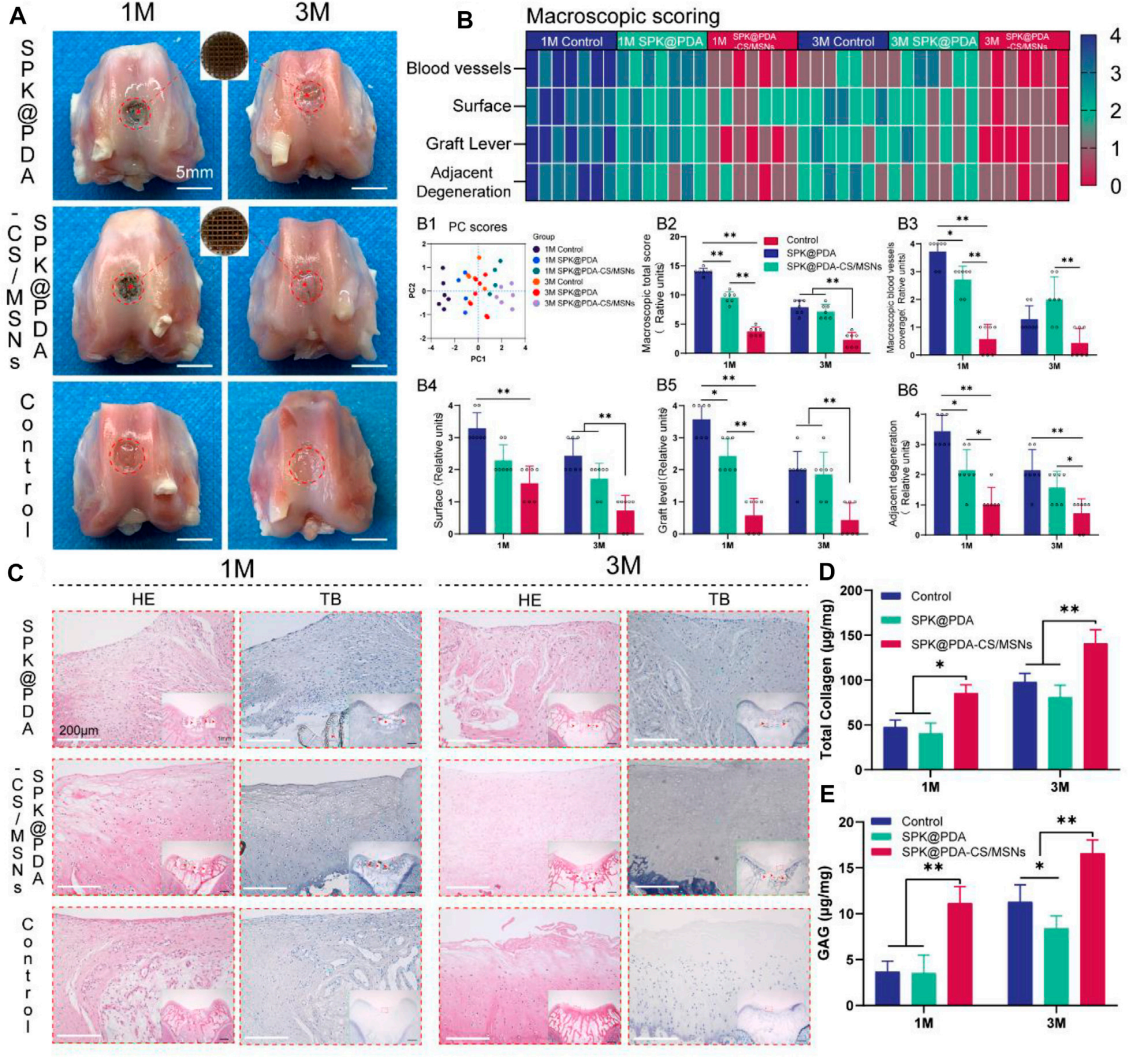
Gross observation and histological analysis of cartilage defects treated with SPK@PDA and SPK@PDA-CS/MSNs scaffolds at 1 and 3 months after surgery. **(A)** Gross morphology of the repaired tissue. **(B)** Heat map of the macroscopic scoring system. B1. Principal component analysis of the macroscopic score. B2. Total macroscopic score comparison of different groups. B3-6. Comparison of variables in different groups, including blood vessel coverage (B3), surface (B4), graft level (B5), and adjacent cartilage degeneration (B6). Data are shown as mean ± SD, *n* = 7, *p < 0.05, **p < .01. **(C)** Hematoxylin-eosin (HE) and toluidine blue (TB) staining of the repaired tissue at 1 and 3 months after surgery, red arrows indicate residual PEEK scaffold. **(D,E)** Total collagen and GAG content of the repaired tissue at 1 and 3 months after surgery. Data are shown as mean ± SD, *n* = 3, *p < 0.05, **p < .01.

To evaluate the effect of the SPK@PDA and SPK@PDA-CS/MSNs scaffolds on cartilage regeneration, we implanted them into a cartilage defect model of rabbits and observed cartilage regeneration at 1 and 3 months after surgery, using gross observation, histology, biochemical assays, and RT-PCR. Macroscopically, there was no obvious inflammatory reaction in any of the SPK@PDA, SPK@PDA-CS/MSNs, and control groups, and some neo-tissue could be seen in the SPK@PDA and SPK@PDA-CS/MSNs groups, while the smoothness of the repair area, graft level, and adjacent cartilage degeneration varied in the SPK@PDA, SPK@PDA-CS/MSNs, and control groups (Figure 6A). We then semi-quantitatively evaluated the repair effect of the cartilage defect treated with the SPK@PDA and SPK@PDA-CS/MSNs scaffolds *via* macroscopic cartilage repair scoring (Figure 6B). As shown in Figure 6 B1-7, the scoring of macroscopic cartilage repair showed a significantly improved repair effect in the SPK@PDA-CS/MSNs group compared to that of the SPK@PDA and control groups. The individual parameters of blood vessel presence, surface, graft level, and adjacent cartilage degeneration of the repair tissue of defects treated with SPK@PDA-CS/MSNs were superior to those of the SPK@PDA and control groups.

**FIGURE 7:**
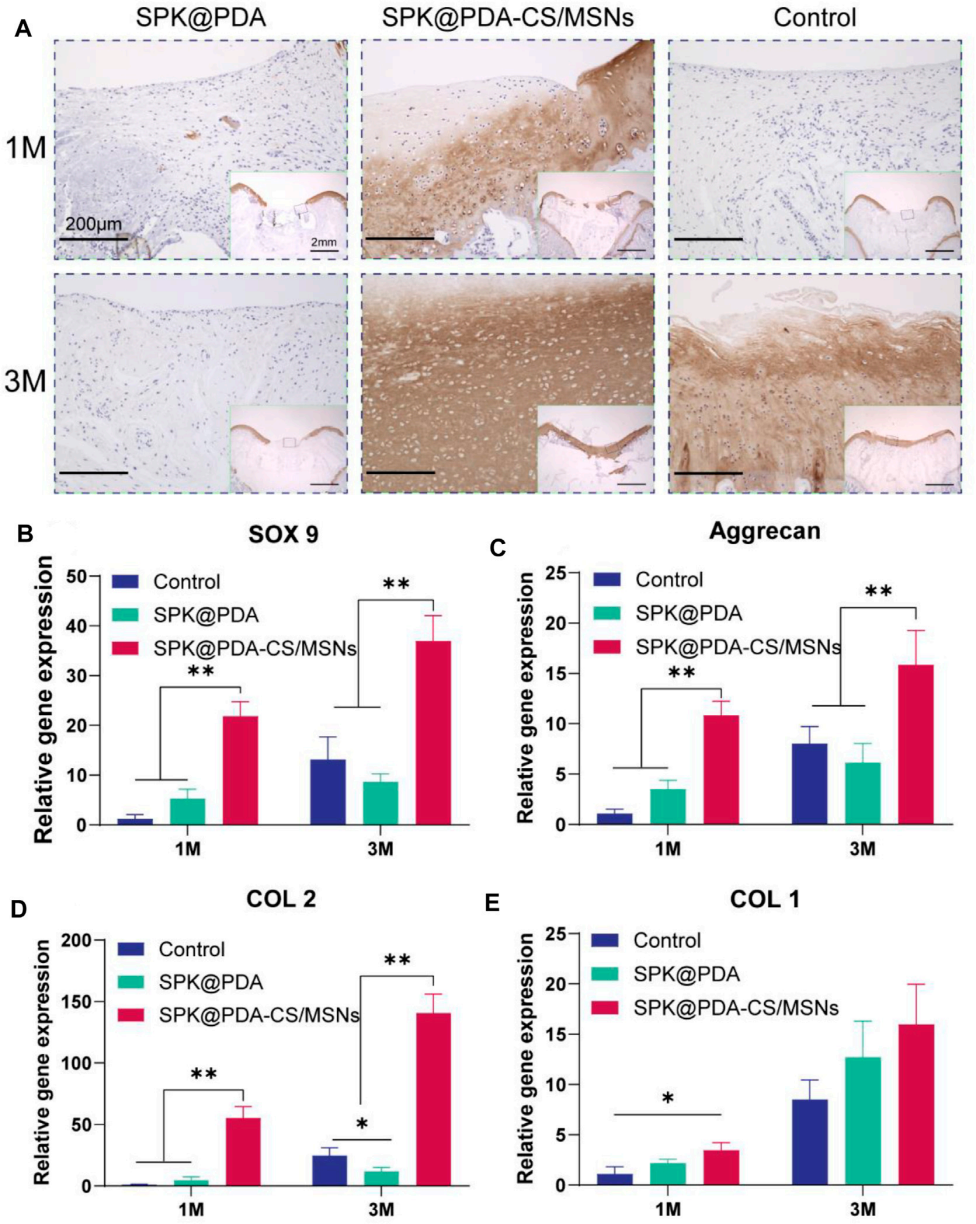
Immunohistochemical staining and RT-PCR analysis for cartilage repair. **(A)** Immunohistochemical staining with collagen II of the repaired tissue at 1 and 3 months after surgery. **(B–E)** Expression of related genes, SOX9 **(B)**, aggrecan **(C)**, collagen II **(D)**, and collagen I **(E)** of the repaired tissue at 1 and 3 months after surgery. Data are showed as mean ± SD, *n* = 3. *p < .05, **p < .01.

The histological findings according to HE and TB staining are showed in Figure 6C, which revealed that application of the SPK@PDA-CS/MSNs scaffold significantly improved the repair effect of the cartilage defect relative to the SPK@PDA and control groups. We then evaluated the total collagen and GAG content of the repair tissue using a biochemical assay, as shown in Figures 6D,E, which revealed that application of the SPK@PDA-CS/MSNs scaffold significantly increased the collagen and GAG contents of the repair tissue relative to the SPK@PDA and control groups, which was consistent with the histological findings. Furthermore, the immunohistochemistry analysis of collagen II also showed that application of the SPK@PDA-CS/MSNs significantly increased collagen II staining intensity compared to the SPK@PDA and control groups at 1 and 3 months after surgery (Figure 7A).

RT-PCR with mRNA isolated from the repair tissue was used to evaluate the expression of cartilage-related genes, including SOX 9 (Figure 7B), aggrecan (Figure 7C), collagen II (Figure 7D), and collagen I (Figure 7E). The gene expression of SOX 9, aggrecan, and collagen II in the SPK@PDA-CS/MSNs group were higher than those in the SPK@PDA and control groups at 1 and 3 months after surgery, respectively, while the collagen I expression of the repair tissue in the SPK@PDA-CS/MSNs group was only higher than that in the control group at 1 month after surgery. Other groups only showed slight changes, which were not statistically significant.

## 4. Discussion

In the present study, we propose a new therapeutic concept for osteoarticular lesions. This is different from the traditional tissue engineering strategy aimed at complete regeneration and the prosthesis replacement strategy aimed at pure functional replacement, because it utilizes a porous prosthesis with regenerative activity. Compared with the traditional “regeneration” and “replacement” strategies, the porous prosthetic strategy with induced regenerative activity may have two major advantages, one being its faster functional recovery ability that can quickly accommodate the needs of patients for early functional recovery compared to the regeneration strategy; the second being its ability to promote partial cartilage biological regeneration to restore the biological friction mechanism of the articular cartilage surface.

In order to evaluate the strategy of porous prostheses with regenerative activity, we first fabricated a drug delivery system based on MSNs and chitosan (CS) using a microfluidic method, which has the ability to sequentially and sustainedly release PDGF-BB and KGN. We evaluated the physicochemical properties and cytotoxicity of different CS/MSNs composite microspheres and chose the CS/MSNs-4 mixture as the drug delivery carrier for PDGF-BB and KGN because of its superior structural characteristics. We then observed the release behavior of PDGF-BB and KGN from CS/MSNs composite microspheres and confirmed that the CS/MSNs delivery system could sequentially and sustainably release PDGF-BB and KGN. This may be due to the extended transport path required for KGN release, which involves diffusion from the MSNs to the CS matrix and travel across the CS matrix, while the PDGF-BB diffuses directly from the CS matrix. Subsequently, we constructed a 3D porous PEEK scaffold following our previous study and performed surface modification on the scaffold through sulfonation treatment, and then immobilized the CS/MSNs composite microspheres (containing PDGF-BB and KGN) in the SPK scaffold through PDA coating to fabricate the SPK@PDA-CS/MSNs scaffold. We investigated the microstructure and hydrophilicity of these scaffolds via SEM and WCA, which demonstrated that the SPK@PDA-CS/MSNs scaffold had an excellent pore structure and a hydrophilic surface. We then evaluated the cytocompatibility of the prepared scaffolds and confirmed that the SPK@PDA-CS/MSNs scaffold could promote BMSC adhesion, spread, and growth, and was biocompatible. We assessed the biofunctionalities of the scaffolds for cell chemotactic ability and chondrogenic differentiation capacity through the transwell system, and the results confirmed that the SPK@PDA-CS/MSNs scaffold (containing PDGF-BB and KGN) had the capacity to recruit BMSCs and promote chondrogenic differentiation of BMSCs.

In this study, we successfully fabricated a porous bioactive prosthesis with excellent biocompatibility and superior biofunctionalities for BMSC recruitment and chondrogenic differentiation capacity *in vitro*. We then implanted the scaffold into the cartilage defect model of rabbits and evaluated its feasibility and safety at 1 and 3 months after surgery. The gross observations and histological results confirmed that the SPK@PDA-CS/MSNs scaffold could effectively fill the cartilage defects and promote biological regeneration of the cartilage located inside and on the surface of the porous scaffold, which may have the potential to restore the biological lubrication of the articular cartilage surface. We then quantitatively analyzed the collagen and GAG content of the repaired tissue through biochemical assays and RT-PCR, and these results suggested that the SPK@PDA-CS/MSNs scaffold could promote cartilage regeneration *in vivo*, which was consistent with the histological results.

To the best of our knowledge, this is the first study to propose a new concept of a porous bioactive prosthesis for articular cartilage lesions, and to systematically evaluate the feasibility of the porous bioactive prosthesis with induced regeneration activity for articular cartilage damage repair. Of course, this was only a relatively elementary study, and there were some limitations and drawbacks. First, the animal model was not optimal, as the regenerative potential of rabbits was greater than that of large animals, such as sheep, which should be investigated in a future study. Second, we did not present the tribological properties of the porous scaffold, which may not have a high coefficient of friction under compressive stress and lubrication of the joint fluid. Third, we did not assess the functional and tribological properties of the repaired tissue in this study, although it would be difficult to perform in rabbits, it would be meaningful for assessing the feasibility of the porous prostheses, and it will be performed in our next large animal experiment. Finally, this was only a preliminary study on the new therapeutic concept for cartilage lesions, and before clinical application can occur, many *in vitro* and *in vivo* experiments are needed to further verify the feasibility and safety of this therapeutic concept.

**FIGURE 8:**
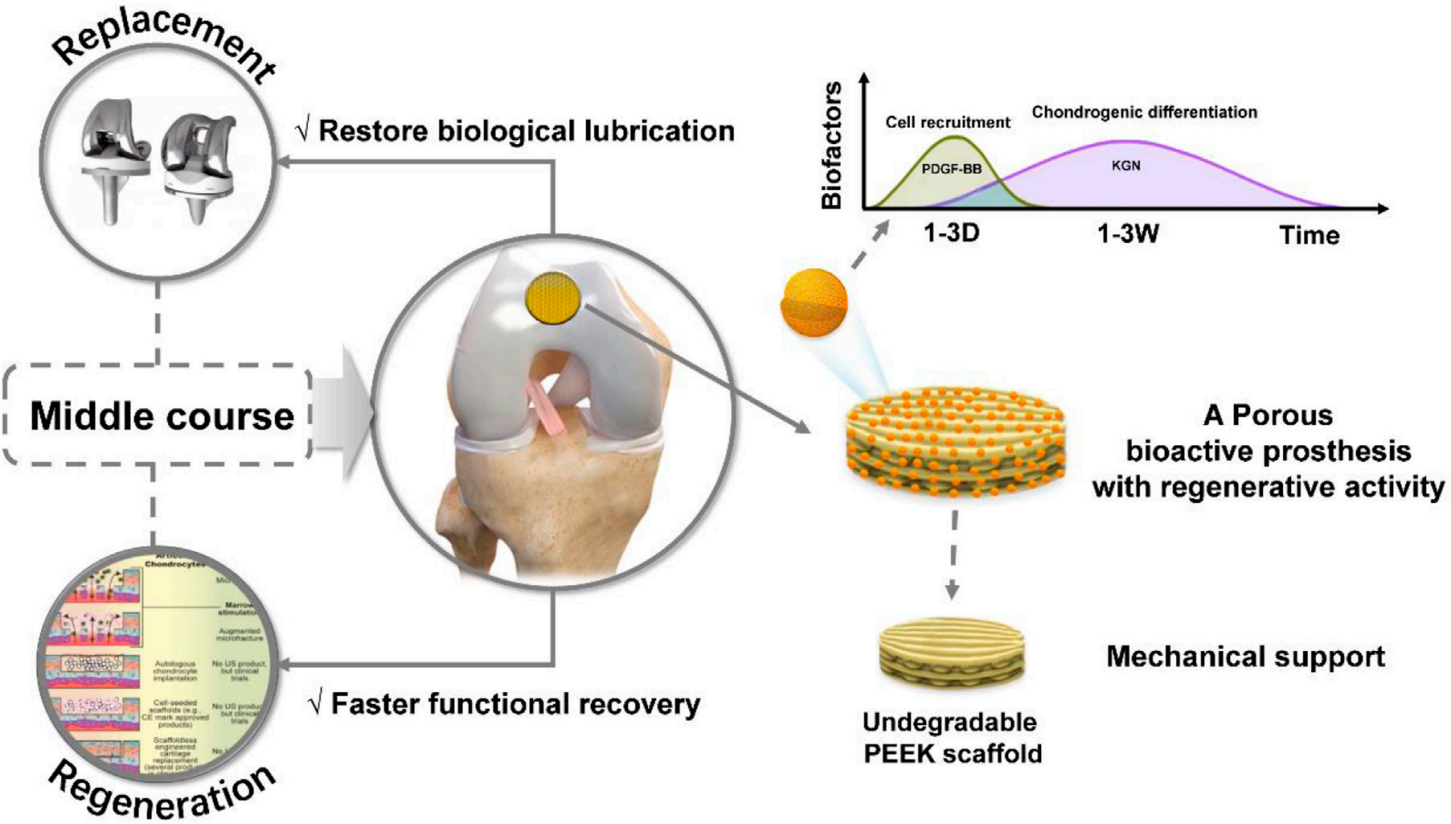
Schematic diagram of the porous bioactive prosthesis with regenerative activity as an intermediate route between “replacement” and “regeneration” for cartilage injury repair.

In this study, as shown in Figure 8, we proposed a porous bioactive prosthesis strategy as a new middle course between “replacement” and “regeneration” for osteoarticular lesions. To achieve the bioactivity of the porous prosthesis in inducing cartilage regeneration, we fabricated a drug delivery system using a microfluidic chip, which could sequentially and sustainably release a cell chemotactic factor, PDGF-BB, and a chondrogenic differentiation cytokine, KGN, and then integrated the drug carrier and a porous PEEK scaffold to construct a porous bioactive prosthesis with regenerative activity. We also evaluated the feasibility of using a porous bioactive prosthesis for cartilage lesions and found that the bioactive prosthesis could induce cartilage regeneration to promote fast functional recovery and restore the biological friction mechanism of the articular cartilage surface. In view of the difficulty in achieving complete cartilage regeneration and the side effects of joint replacement, the porous bioactive prosthesis strategy may become a new treatment for cartilage injury in the near future.

## 5 Conclusion

In this study, we first fabricated a drug delivery system (CS/MSNs composite microspheres loaded with PDGF-BB and KGN) based on MSNs and CS using a microfluidic chip, which could sequentially and sustainably release PDGF-BB and KGN. Then, we integrated the CS/MSNs composite microspheres loaded with PDGF-BB and KGN and a 3D printed porous PEEK scaffold to construct the SPK@PDA-CS/MSNs scaffold, a porous bioactive prosthesis with regenerative activity. We systematically evaluated the biocompatibility and biofunctionality of the SPK@PDA-CS/MSNs scaffold through *in vitro* and *in vivo* experiments and confirmed that the SPK@PDA-CS/MSNs scaffold was biocompatible, enhanced chondrogenic differentiation of BMSCs *in vitro*, and promoted cartilage regeneration *in vivo*. The application of a porous bioactive prosthesis may represent a new treatment for cartilage injury.

## Data Availability

The original contributions presented in the study are included in the article/Supplementary Material, further inquiries can be directed to the corresponding author.
