# Supplementary material for: Porous Bioactive Prosthesis With Chitosan/Mesoporous Silica Nanoparticles Microspheres Sequentially and Sustainedly Releasing Platelet-Derived Growth Factor-BB and Kartogenin: A New Treatment Strategy for Osteoarticular Lesions
Source: Front Bioeng Biotechnol. 2022 Feb 3;10:839120. doi: 10.3389/fbioe.2022.839120 (PMC8850694; doi:10.3389/fbioe.2022.839120)
Supplement: Supplementary file 1 [file DataSheet1.docx]

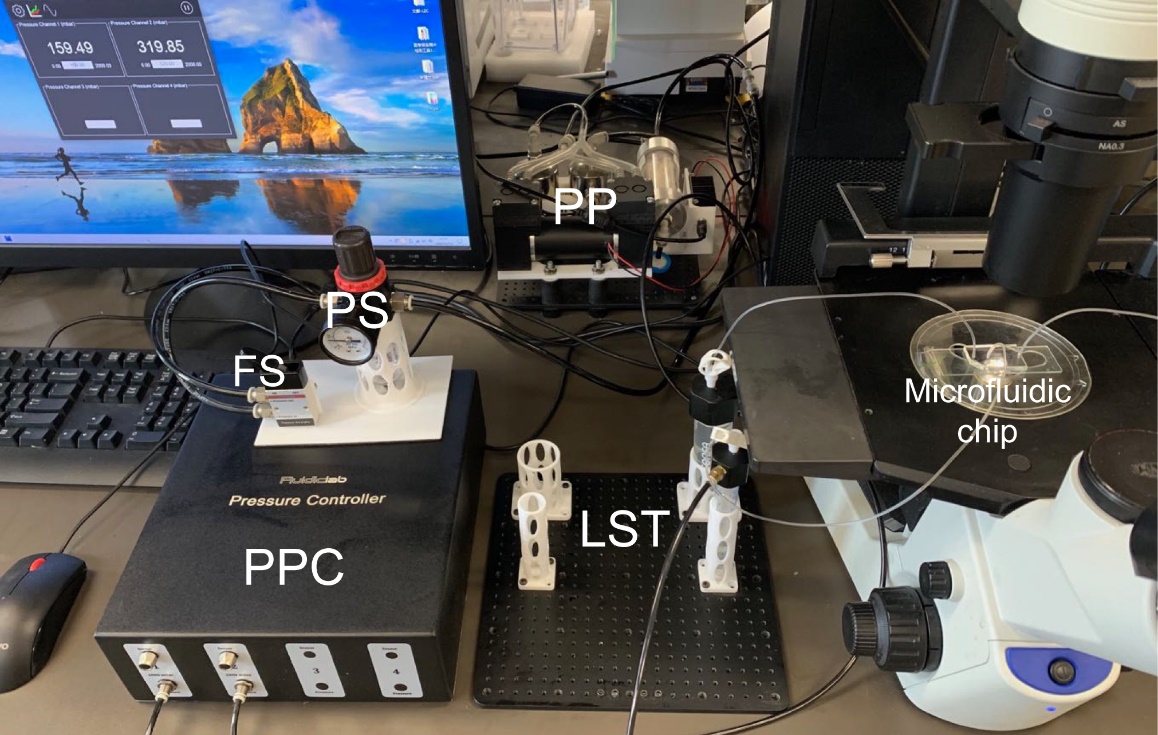


Figure S1. The microfluidic instrumentation used in this study.


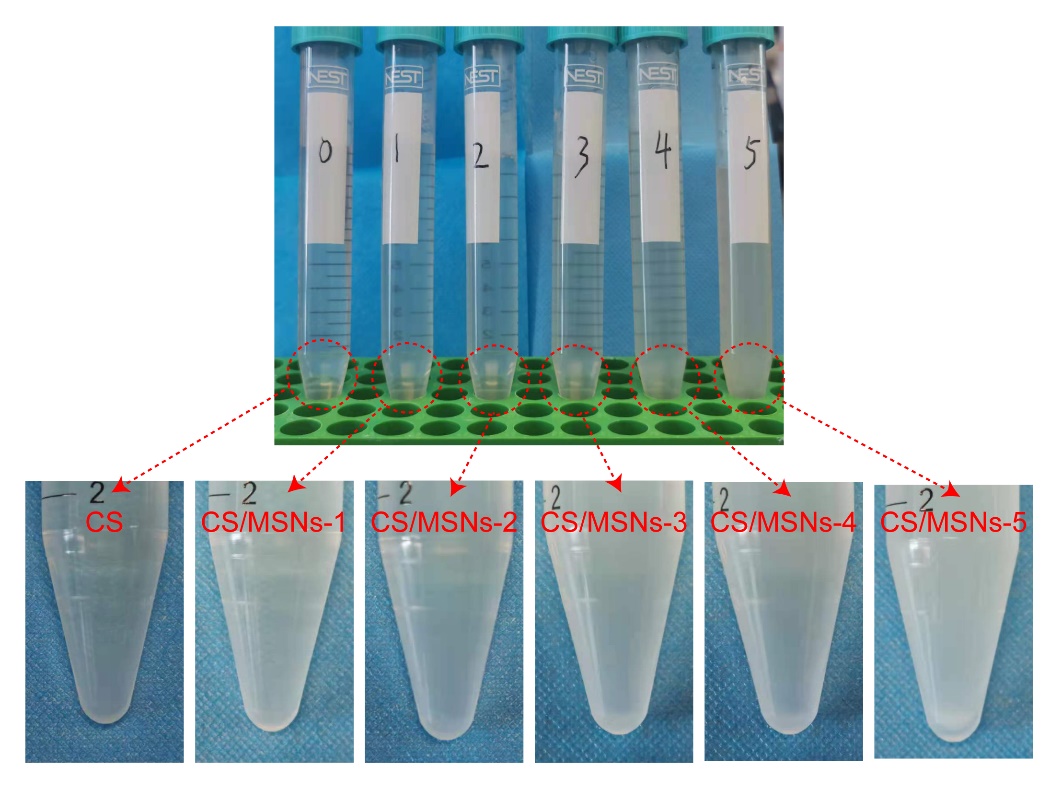


Figure S2. The gross observations of the CS/MSNs solution.


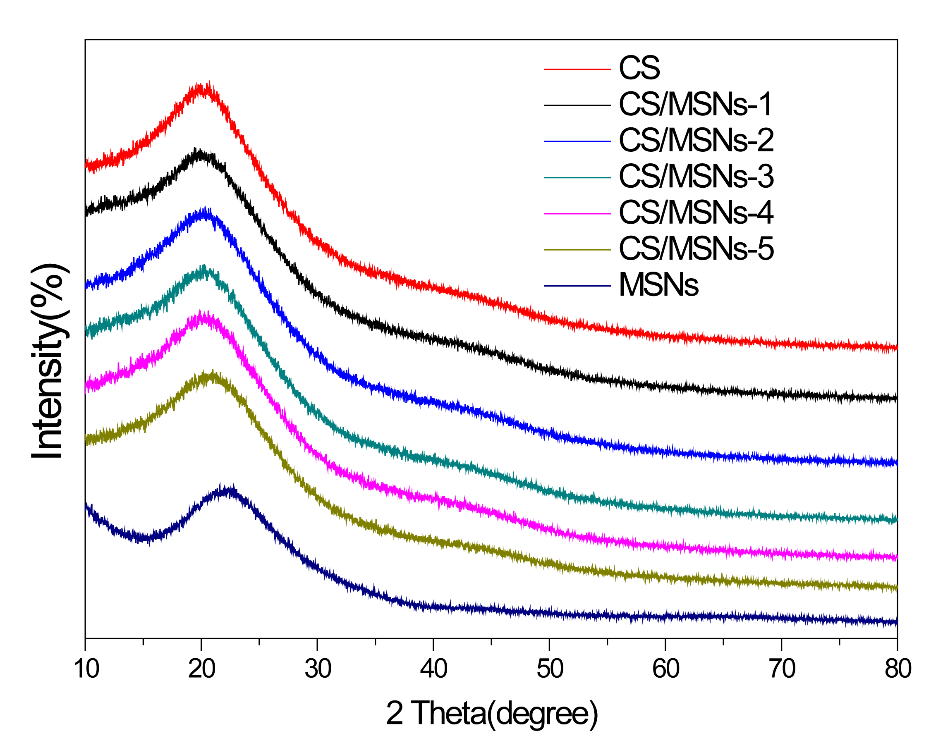


Figure S3. The XRD patterns of CS, CS/MSNs, and MSNs.


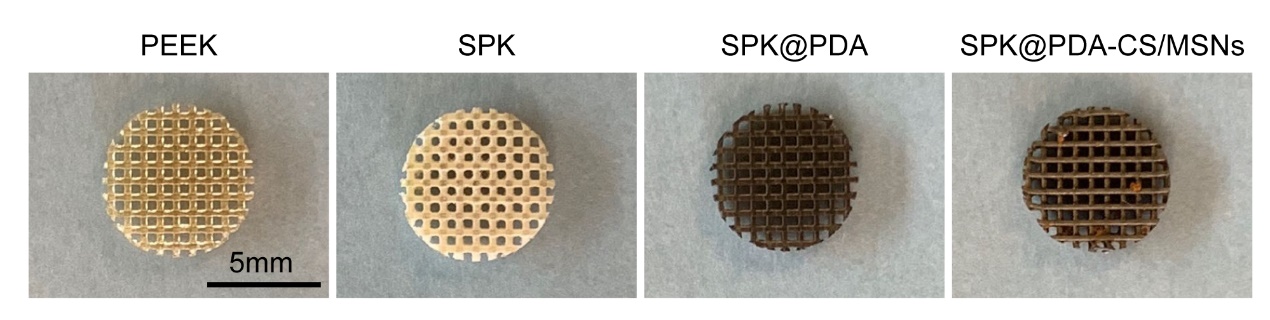


Figure S4. The macroscopic observation of the prepared scaffolds, PEEK, SPK, SPK@PDA and SPK@PDA-CS/MSNs.


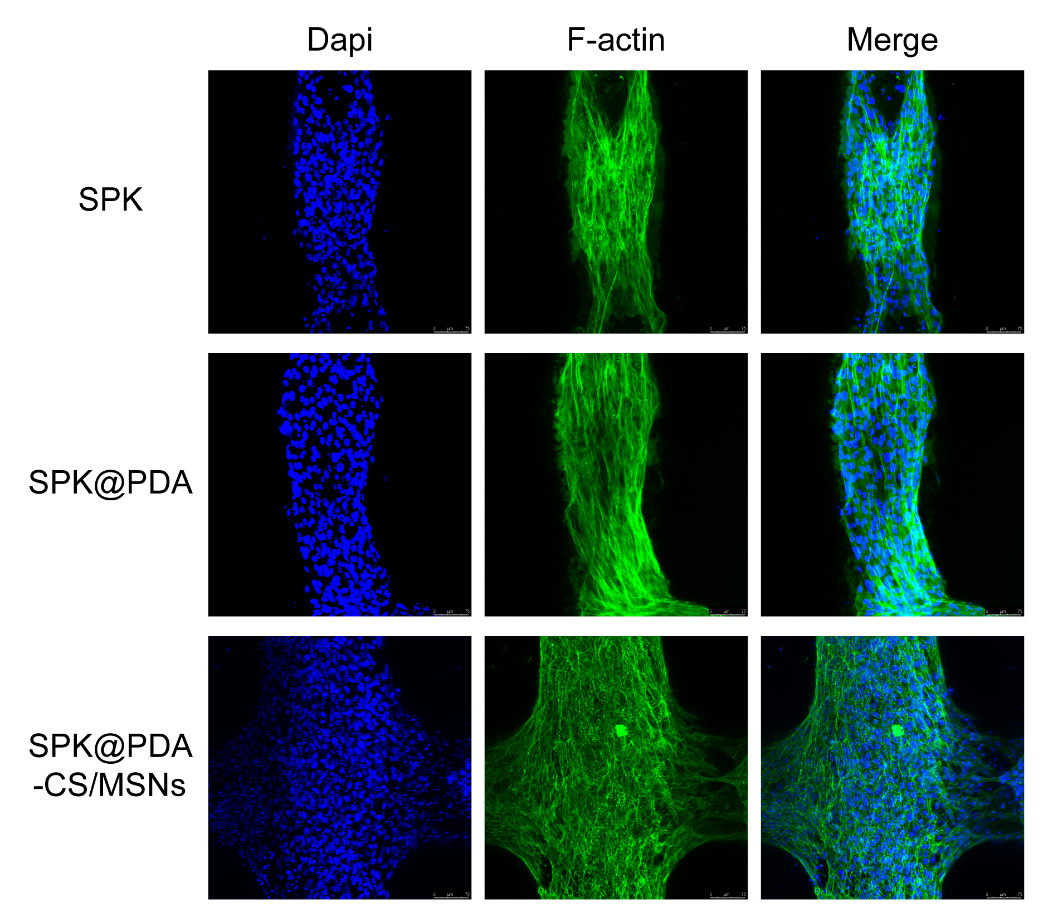


Figure S5. The cytoskeleton staining of BMSCs on the SPK, SPK@PDA and SPK@PDA-CS/MSNs scaffolds for 3 days of culture.

Table S1. The primers used in this study

| **Gene** | **Primer** |
| --- | --- |
|  |  |
| SOX 9 | Forward: GCGGAGGAAGTCGGTGAAGAAT |
|  | Reverse AAGATGGCGTTGGGCGAGAT |
| Col II | Forward CACGCTCAAGTCCCTCAACA |
|  | Reverse TCTATCCAGTAGTCACCGCTCT |
| Aggrecan | Forward GGAGGAGCAGGAGTTTGTCAA |
|  | Reverse TGTCCATCCGACCAGCGAAA |
| Col I | Forward GCCACCTGCCAGTCTTTACA |
|  | Reverse CCATCATCACCATCTCTGCCT |
| GAPDH | Forward CAAGAAGGTGGTGAAGCAGG |
|  | Reverse CACTGTTGAAGTCGCAGGAG |

Table S2.

| Newly developed macroscopic scoring system | | |
| --- | --- | --- |
| Parameter | Item | Points |
| Presence of blood vessels in the repair tissue | No | 0 |
|  | Less than 25% of the repair tissue | 1 |
|  | 25-50% of the repair tissue | 2 |
|  | 50-75% of the repair tissue | 3 |
|  | More than 75% of the repair tissue | 4 |
| Surface of the repair tissue | Smooth, homogeneous | 0 |
|  | Smooth, heterogeneou | 1 |
|  | Fibrillated | 2 |
|  | Incomplete new repair tissue | 3 |
|  | No repair tissue | 4 |
| Graft level with surrounding cartilage | In level with adjacent cartilage | 0 |
|  | Raised or Below <25% repair of defect depth | 1 |
|  | Raised or Below ≥25% ＜50% repair of defect depth | 2 |
|  | Raised or Below≥50% ＜75% repair of defect depth | 3 |
|  | Raised or Below≥75% repair of defect depth | 4 |
| Degeneration of adjacent articular cartilage | Normal | 0 |
|  | Cracks and/or fibrillations in integration zone | 1 |
|  | Diffuse osteoarthritic changes | 2 |
|  | Extension of the defect into the adjacent cartilage | 3 |
|  | Subchondral bone damage | 4 |

A total number of 16 points is achieved for the worst possible result.
